# Clean Self-Supervised MRI Reconstruction from Noisy, Sub-Sampled Training Data with Robust SSDU

**DOI:** 10.3390/bioengineering11121305

**Published:** 2024-12-23

**Authors:** Charles Millard, Mark Chiew

**Affiliations:** 1 Wellcome Centre for Integrative Neuroimaging, FMRIB, University of Oxford, Oxford OX3 9DU, UK; 2 Department of Medical Biophysics, University of Toronto, Toronto, ON M4N 3M5, Canada; 3 Physical Sciences, Sunnybrook Research Institute, Toronto, ON M4N 3M5, Canada

**Keywords:** deep learning, image reconstruction, magnetic resonance imaging

## Abstract

Most existing methods for magnetic resonance imaging (MRI) reconstruction with deep learning use fully supervised training, which assumes that a fully sampled dataset with a high signal-to-noise ratio (SNR) is available for training. In many circumstances, however, such a dataset is highly impractical or even technically infeasible to acquire. Recently, a number of self-supervised methods for MRI reconstruction have been proposed, which use sub-sampled data only. However, the majority of such methods, such as Self-Supervised Learning via Data Undersampling (SSDU), are susceptible to reconstruction errors arising from noise in the measured data. In response, we propose Robust SSDU, which provably recovers clean images from noisy, sub-sampled training data by simultaneously estimating missing k-space samples and denoising the available samples. Robust SSDU trains the reconstruction network to map from a further noisy and sub-sampled version of the data to the original, singly noisy, and sub-sampled data and applies an additive Noisier2Noise correction term upon inference. We also present a related method, Noiser2Full, that recovers clean images when noisy, fully sampled data are available for training. Both proposed methods are applicable to any network architecture, are straightforward to implement, and have a similar computational cost to standard training. We evaluate our methods on the multi-coil fastMRI brain dataset with novel denoising-specific architecture and find that it performs competitively with a benchmark trained on clean, fully sampled data.

## 1. Introduction

Magnetic resonance imaging (MRI) has excellent soft tissue contrast and is the gold standard modality for a number of clinical applications. A hindrance of MRI, however, is its lengthy acquisition time, which is especially challenging when high spatio-temporal resolution is required, such as for dynamic imaging [1]. To address this, there has been substantial research attention on methods that reduce the acquisition time without significantly sacrificing the diagnostic quality [2,3,4]. In MRI, measurements are acquired in the Fourier representation of the image, referred to in the MRI literature as “k-space”. Since the acquisition time is roughly proportional to the number of k-space samples, acquisitions can be accelerated by sub-sampling. A reconstruction algorithm is then employed to estimate the image from the sub-sampled data.

In recent years, reconstructing sub-sampled MRI data with neural networks has emerged as a state-of-the-art method [5,6,7]. The majority of existing methods assume that a fully sampled dataset is available for fully supervised training. However, for many applications, no such dataset is available and may be difficult or even infeasible to acquire in practice [8,9,10]. In response, there have been a number of self-supervised methods proposed, which train on sub-sampled data only [11,12,13,14]. Many such methods have shown promise in a broad range of clinical applications where fully sampled data are challenging to acquire, including dynamic imaging [15], late gadolinium enhancement cardiac imaging [16], simultaneous multi-slice functional imaging [17], and multi-contrast imaging [18].

Most existing training methods assume that the measurement noise is small and does not explicitly denoise sampled data. Section 3 shows theoretically that without explicit denoising, the reconstruction quality degrades when the measurement noise increases. This is a particular concern for low SNR measurements, where the SNR is the ratio of the signal and noise amplitudes, and the SNR is considered “low” when the measurement noise contributes substantially to the difference between the noisy, sub-sampled data and the ground truth. For instance, the data acquired from low-cost, low-field scanners are considered having a low SNR [19,20,21].

The goal of this paper is to develop a theoretically rigorous, computationally efficient approach for simultaneous self-supervised reconstruction and denoising that performs comparably to fully supervised training. The primary challenge of this goal is that many existing self-supervised denoising methods are not applicable to data that are also sub-sampled [22], depend on paired instances of noisy data [23], or are substantially computationally more expensive than fully supervised learning in training time [24].

This paper proposes a modification of Self-Supervised Learning via Data Undersampling (SSDU) [13] that also removes measurement noise, building on the present authors’ recent work [25] on the connection between SSDU and the multiplicative version of the self-supervised denoising method Noisier2Noise [26]. Our method, which we term “Robust SSDU”, combines SSDU with the *additive* Noisier2Noise. In brief, Robust SSDU trains a network to map from a further sub-sampled and further noisy version of the training data to the original sub-sampled, noisy data. Then, upon inference, a correction is applied to the network output that ensures that the clean (i.e., noise-free) image is recovered as expected.

We find that Robust SSDU performs competitively with a fully supervised benchmark where the network is trained on clean, fully sampled data, despite training on noisy, sub-sampled data only. We also propose a related method that recovers clean images for the simpler task of noisy data being available for training when fully sampled, which we term “Noisier2Full”. Both Noisier2Full and Robust SSDU are fully mathematically justified and have minimal additional computational expenses compared to standard training.

The existing method most similar to Robust SSDU is Noise2Recon-Self-Supervised (Noise2Recon-SS) [27]. The proposed method, Robust SSDU, has a number of key difference to Noise2Recon-SS, including a loss weighting and an additive Noisier2Noise correction term upon inference that statistically guarantees the recovery of the ground truth; see Section 4.3 for a detailed comparison. To our knowledge, Robust SSDU is the first method that provably recovers clean images when only noisy, randomly sub-sampled data are available for training. In practice, we find that Robust SSDU offers substantial image quality improvements over Noise2Recon-SS and a two-fold reduction in computational cost during training; see Section 5.

### Notation

This paper uses notation consistent with [25]. We use the subscripts *t* and *s* to index the training set T and test set S, respectively. For instance, data in the training and test set are denoted by $y_{t}$ and $y_{s}$, respectively. Random variables are represented as their instances without indices and are capitalized if they are vectors. For instance, $y_{t},y_{s}∽Y$ for vectors and $M_{\Omega_{t}},\;M_{\Omega_{s}}∽M_{\Omega }$ for matrices, where ∽ denotes that the left-hand side is an instance of the random variable on the right-hand side.

We use $Y_{0}$ to refer to the ground truth, *Y* to refer to the data, $\tilde{Y}$ to refer to the further corrupted data, and $\hat{Y}$ to refer to an estimate of the ground truth. We note that Section 2.1, Section 2.2, and Section 3 onward discuss different recovery tasks, so the definitions of, for instance, the data, *Y*, and their instances are section-specific.

## 2. Theory: Background

Image recovery with deep learning is a regression problem, so it is centered around the conditional distribution $Y_{0}|Y$, where $Y_{0}$ and *Y* are the random variables associated with the ground truth and data, respectively [28]. If the ground truth data $y_{0,t}∽Y_{0}$ are available for training, fully supervised learning can be employed to characterize $Y_{0}|Y$ directly [29]. This paper focuses on self-supervised learning, which concerns the task of training a network to estimate the ground truth when the training data are $y_{t}∽Y$ so are themselves corrupted [23,24,30,31].

The remainder of this section reviews key works from the self-supervised learning literature that form the bases of the methods proposed in this paper. Section 2.1 presents the case where the data corruption is Gaussian noise, and Section 2.2 presents the case where the data corruption is sub-sampling.

### 2.1. Self-Supervised Denoising with Noisier2Noise

Denoising with deep learning aims to recover a clean *q*-dimensional vector from noisy data:

(1) $$\begin{array}{c} y_{s}=y_{0,s}+n_{s}, \end{array}$$

where $n_{s}$ is noise and s∈S indexes the test set. In MRI, noise in k-space is modeled as a complex zero-mean Gaussian, $n_{s}∽\mathrm{CN}\left(0,\Sigma_{n}^{2}\right)$, where $\Sigma_{n}^{2}$ is a covariance matrix that can be estimated, for instance, with an empty pre-scan [32]. We treat the noise as white, $\Sigma_{n}^{2}=\sigma_{n}^{2}1$, noting that noise with non-trivial covariance can be whitened by left-multiplying $y_{s}$ with the square root inverse of $\Sigma_{n}^{2}$, denoted by $\Sigma_{n}^{-1}$. Other noise distributions are discussed in Section 6.

This paper focuses on the additive Noisier2Noise [26] because we find that it offers a natural way to extend image reconstruction to low-SNR data; see Section 3. Noisier2Noise’s training procedure consists of corrupting noisy training data with further noise and training a network to recover a singly noisy image from a noisier image. Concretely, for each $y_{t}$, further noise is introduced:

(2) $$\begin{array}{c} {\tilde{y}}_{t}=y_{t}+{\tilde{n}}_{t}=y_{0,t}+n_{t}+{\tilde{n}}_{t}, \end{array}$$

where ${\tilde{n}}_{t}∽\mathrm{CN}\left(0,\alpha^{2}\sigma_{n}^{2}1\right)$ for a constant α. Then, a network $f_{\theta }$ with parameters θ is trained to minimize the sum

(3) $$\begin{array}{c} \hat{\theta }=\underset{\theta }{\arg\min}\sum_{t\in T}{∥f_{\theta }\left({\tilde{y}}_{t}\right)-y_{t}∥}_{2}^{2} \end{array}$$

where the symbol ∑ is used exclusively for summation herein. The following result states that a simple transform of the trained network yields the ground truth in expectation despite never seeing the ground truth during training. Here, and throughout this paper, expectations are taken over all random variables.

**Result 1.**  *Consider the random variables $Y=Y_{0}+N$ and $\tilde{Y}=Y+\tilde{N}$, where N and $\tilde{N}$ are zero-mean Gaussians distributed with variances of $\sigma_{n}^{2}$ and $\alpha^{2}\sigma_{n}^{2}$, respectively. Minimizing*

(4) $$\theta *=\underset{\theta }{\arg\min}\;E[∥f_{\theta }\left(\tilde{Y}\right){-Y∥}_{2}^{2}\left|\tilde{Y}\right]$$

*yields a network that satisfies*

(5) $$E\left[Y_{0}|\tilde{Y}\right]=\frac{\left(1+\alpha^{2}\right)f_{\theta *}\left(\tilde{Y}\right)-\tilde{Y}}{\alpha^{2}}.$$

**Proof.**  See Section 3.3 of [26]. □

Here, Equation (4) can be thought of as Equation (3) in the limit of an infinite number of samples and $\hat{\theta }$ as a finite sample approximation of θ*. Result 1 states that a clean image can be estimated in a conditional expectation by employing a correction term based on α. It suggests the following procedure for estimating $y_{0,s}$ upon inference: corrupt the test data $y_{s}$ with further noise, ${\tilde{y}}_{s}=y_{s}+{\tilde{n}}_{s}$; apply the trained network to the further noisy data, $f_{\hat{\theta }}\left({\tilde{y}}_{s}\right)$; and correct the output using the right-hand side of Equation (5).

### 2.2. Self-Supervised Reconstruction with SSDU

This section focuses on the case where the data consist of noise-free, sub-sampled data:

(6) $$\begin{array}{c} y_{s}=M_{\Omega_{s}}y_{0,s}. \end{array}$$

Here, $M_{\Omega_{s}}$ is a sampling mask, a diagonal matrix with a *j*th diagonal of 1 when $j\in \Omega_{s}$ and otherwise 0 for the sampling set $\Omega_{s}\subseteq \left\{1,2,\ldots ,q\right\}$.

Self-supervised reconstruction consists of training a network to recover images when only sub-sampled data is available for training: $y_{t}=M_{\Omega_{t}}y_{0,t}$[33]. This work focuses on the popular method SSDU [13], which was theoretically justified in [25] via the multiplicative noise version of Noiser2Noise [26]. In this framework, analogous to the further noise used in Equation (2), the training data $y_{t}$ are *further sub-sampled* by applying a second mask with the sampling set $\Lambda_{t}\subseteq \left\{1,2,\ldots ,q\right\}$ to $y_{t}$:

(7) $$\begin{array}{c} {\tilde{y}}_{t}=M_{\Lambda_{t}}y_{t}=M_{\Lambda_{t}\cap \Omega_{t}}y_{0,t}, \end{array}$$

where $M_{\Lambda_{t}\cap \Omega_{t}}=M_{\Lambda_{t}}M_{\Omega_{t}}$. Training consists of minimizing a loss function on indices in $\Omega_{t}\setminus \Lambda_{t}$, such as

(8) $$\hat{\theta }=\underset{\theta }{\arg\min}\sum_{t\in T}{∥M_{\Omega_{t}\setminus \Lambda_{t}}\left(f_{\theta }\left({\tilde{y}}_{t}\right)-y_{t}\right)∥}_{2}^{2},$$

where $M_{\Omega_{t}\setminus \Lambda_{t}}=\left(1-M_{\Lambda_{t}}\right)M_{\Omega_{t}}$. Although for theoretical ease we state SSDU with an $\ell_{2}$ loss here, it is known that other losses are possible [13].

Let $p_{j}=P\left[j\in \Omega \right]$ and ${\tilde{p}}_{j}=P\left[j\in \Lambda \right]$. Assuming that

(9) $$\begin{array}{cc} p_{j} & >0\;\forall \;j, \end{array}$$

(10) $$\begin{array}{ll} {\tilde{p}}_{j}<1 & \;\forall \;\{j:p_{j}<1\}, \end{array}$$

the following result from [25] proves that SSDU recovers the clean image as expected.

**Result 2.**  *Consider the random variables $Y=M_{\Omega }Y_{0}$ and $\tilde{Y}=M_{\Lambda }Y$. When Equations (9) and (10) hold, minimizing*

(11) $$\theta *=\underset{\theta }{\arg\min}\;E[∥M_{\Omega \setminus \Lambda }\left(f_{\theta }\left(\tilde{Y}\right)-Y\right){∥}_{2}^{2}\left|\tilde{Y}\right]$$

*yields a network with parameters that satisfies*

(12) $$M_{{\left(\Lambda \cap \Omega \right)}^{c}}E\left[Y_{0}|\tilde{Y}\right]=M_{{\left(\Lambda \cap \Omega \right)}^{c}}f_{\theta *}\left(\tilde{Y}\right).$$

**Proof.**  See Appendix B of [25] (where [25] uses $1-M_{\Lambda }M_{\Omega }$, this uses paper the more compact notation $M_{{\left(\Lambda \cap \Omega \right)}^{c}}$, where superscript *c* denotes the complement of a set). □

Result 2 states that the network correctly estimates $Y_{0}$ in a conditional expectation for indices not in Λ∩Ω. To estimate everywhere in k-space, one can overwrite sampled indices or use data-consistent architecture; see [25] for details.

## 3. Theory: Proposed Methods

The remainder of this paper considers the task of training a network to recover images from data that are both noisy *and* sub-sampled:

(13) $$\begin{array}{c} y_{s}=M_{\Omega_{s}}\left(y_{0,s}+n_{s}\right). \end{array}$$

It has been stated that when a network reconstructs noisy MRI data with a standard training method, there is a denoising effect [20]. In the following, we are motivated by the need for methods that explicitly remove noise by showing that the apparent noise removal is in fact a “pseudo-denoising” effect due to the correct estimation of the ground truth in an expectation only for indices in $\Omega^{c}$.

Consider the standard approach of training a network to map from noisy, sub-sampled $y_{t}$ to noisy, fully sampled $y_{0,t}+n_{t}$. In terms of random variables, training consists of minimizing

(14) $$\theta *=\underset{\theta }{\arg\min}\;E[∥f_{\theta }\left(Y\right)-\left(Y_{0}+N\right){∥}_{2}^{2}\left|Y\right],$$

which gives a network that satisfies

(15) $$f_{\theta *}\left(Y\right)=E\left[Y_{0}+N|Y\right].$$

Equation (15) does not hold for completely arbitrary network architecture. The conditions on $f_{\theta }$ (which are also required for Results 1 and 2) are detailed in Section II-A of [25]. In brief, the Jacobian matrix *J* with the entries $J_{ij}=\partial f_{\theta }{\left(Y\right)}_{j}/\partial \theta_{i}$ must have maximally linearly independent rows, which is expected for well-constructed architectures when the number of parameters exceeds *q*. Throughout the remainder of this paper, we assume that $f_{\theta }$ satisfies this condition. We also assume that the optimizer is not stuck in a poor local minimum so that the network is a good approximation of Equation (15) in practice.

It is instructive to examine how $E[Y_{0}+N|Y]$ depends on the sampling mask Ω. Firstly, for j∉Ω,

(16) $$\begin{array}{ll} E[Y_{0,j}+N_{j}|Y,j\notin \Omega ] & =E\left[Y_{0,j}|Y\right]+E\left[N_{j}\right]\\ & =E[Y_{0,j}|Y], \end{array}$$

where we use the independence of $N_{j}$ from *Y* when j∉Ω and $E[N_{j}]=0$ by assumption. For the alternative, j∈Ω,

(17) $$\begin{array}{c} E\left[Y_{0,j}+N_{j}|Y,j\in \Omega \right]=E\left[Y_{j}|Y\right]=Y_{j} \end{array}$$

where $Y_{0,j}+N_{j}=Y_{j}$ for j∈Ω is used. The trained network therefore satisfies

(18) $$\begin{array}{c} f_{\theta *}\left(Y\right)=E\left[Y_{0}+N|Y\right]=M_{\Omega^{c}}E\left[Y_{0}|Y\right]+M_{\Omega }Y. \end{array}$$

Therefore, the network targets the noise-free $Y_{0}$ in regions in $\Omega^{c}$ but recovers the noisy *Y* otherwise. As there is less total measurement noise present than $Y_{0}+N$, this gives the impression of noise removal; however, we emphasize that the network does not remove the noise in *Y*. Since the term “denoising” typically refers to the removal of noise *from the input data*, we use the term “pseudo-denoising” to refer to the behavior stated in Equation (18). Other than the conditions on $f_{\theta }$ described above, this result is agnostic to the network architecture so includes “unrolled” approaches that may have a regularization parameter which is designed to trade off the model and consistency with the data.

We refer to this method described in this section as “Supervised w/o denoising” throughout this paper. In the following, we propose methods that explicitly recover $Y_{0}$ in a conditional expectation from noisy, sub-sampled inputs in two cases: (A) the training data is noisy and fully sampled; (B) the training data is noisy and sub-sampled. For tasks A and B, we propose “Noisier2Full” and “Robust SSDU”, respectively.

### 3.1. Noisier2Full for Fully Sampled, Noisy Training Data

This section proposes Noisier2Full, which extends the additive Noisier2Noise to reconstruction tasks for noisy, fully sampled training data. Based on Equation (2), we propose corrupting the measurements $y_{t}$ with further noise on the sampled indices:

(19) $$\begin{array}{c} {\tilde{y}}_{t}=y_{t}+M_{\Omega_{t}}{\tilde{n}}_{t}. \end{array}$$

Then, we minimize the loss between ${\tilde{y}}_{t}$ and the noisy, fully sampled training data $y_{0,t}+n_{t}$. In terms of random variables,

(20) $$\theta *=\underset{\theta }{\arg\min}\;E[∥f_{\theta }\left(\tilde{Y}\right)-\left(Y_{0}+N\right){∥}_{2}^{2}\left|\tilde{Y}\right].$$

Minimizing the $\ell_{2}$ norm gives a network that satisfies

(21) $$\begin{array}{c} f_{\theta *}\left(\tilde{Y}\right)=E\left[Y_{0}+N|\tilde{Y}\right], \end{array}$$

which is recognizable as Equation (15) with *Y* replaced by $\tilde{Y}$. Similarly to Equation (16), $N_{j}$ is independent of $\tilde{Y}$ when j∉Ω, so the ground truth is estimated in such regions:

(22) $$E\left[Y_{0,j}|\tilde{Y},j\notin \Omega \right]=E\left[Y_{0,j}|\tilde{Y}\right].$$

However, crucially, the expectation is conditional on $\tilde{Y}$, not *Y*, so the additive Noisier2Noise correction stated in Result 1 is applicable when j∈Ω:

(23) $$\begin{array}{c} E\left[Y_{0,j}|\tilde{Y},j\in \Omega \right]=\frac{\left(1+\alpha^{2}\right)f_{\theta *}{\left(\tilde{Y}\right)}_{j}-{\tilde{Y}}_{j}}{\alpha^{2}} \end{array}$$

Although Result 1 is not specifically constructed for sub-sampled data, it is applicable here because it is an entry-wise statistical relationship so can be applied to each index that has the proper noise statistics. Therefore, $Y_{0}$ can be estimated with

(24) $$E\left[Y_{0}|\tilde{Y}\right]=M_{\Omega }\left(\frac{\left(1+\alpha^{2}\right)f_{\theta *}\left(\tilde{Y}\right)-\tilde{Y}}{\alpha^{2}}\right)+M_{\Omega^{c}}f_{\theta *}\left(\tilde{Y}\right).$$

In summary, Noisier2Full recovers $Y_{0}$ in a conditional expectation by introducing further noise to the sampled indices during training and correcting those indices upon inference via additive Noisier2Noise. In the subsequent section, we show how this approach can be extended to the more challenging case where the training data are also sub-sampled.

### 3.2. Robust SSDU for Sub-Sampled, Noisy Training Data

**Figure 1:**
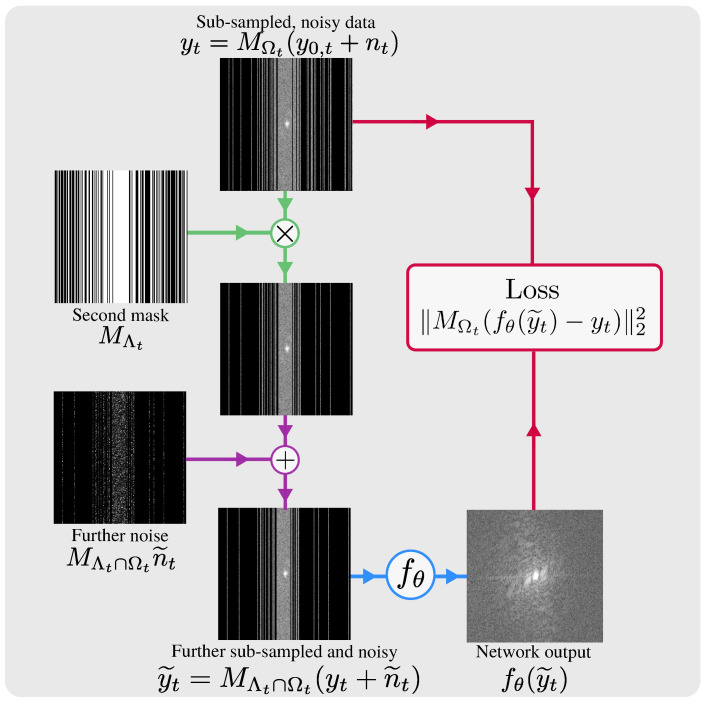
The proposed self-supervised reconstruction and denoising method, Robust SSDU, which extends the training procedure illustrated in Figure 1 of [25] to low-SNR data. The sub-sampled, noisy training data $y_{t}$ are further sub-sampled by a mask $M_{\Lambda_{t}}$ and corrupted by further noise ${\tilde{n}}_{t}$, yielding ${\tilde{y}}_{t}$. The loss is computed between $y_{t}$ and $f_{\theta }\left({\tilde{y}}_{t}\right)$ on $\Omega_{t}$.

This section proposes Robust SSDU, which recovers clean images in a conditional expectation when the training data are both noisy and sub-sampled. Robust SSDU combines the approaches from Section 2.1 and Section 2.2 to simultaneously reconstruct and denoise data; see Figure 1 for a schematic. We propose combining Equations (2) and (7) to form a vector that is further sub-sampled *and* additionally noisy:

(25) $$\begin{array}{c} {\tilde{y}}_{t}=M_{\Lambda_{t}\cap \Omega_{t}}\left(y_{t}+{\tilde{n}}_{t}\right). \end{array}$$

Recall that SSDU employs $M_{\Omega \setminus \Lambda }$ in the loss, which yields a network that estimates indices in ${\left(\Lambda \cap \Omega \right)}^{c}$; see Result 2. For Robust SSDU, we replace $M_{\Omega \setminus \Lambda }$ with $M_{\Omega }$ so that the loss is

(26) $$\hat{\theta }=\underset{\theta }{\arg\min}\sum_{t\in T}{∥M_{\Omega_{t}}\left(f_{\theta }\left({\tilde{y}}_{t}\right)-y_{t}\right)∥}_{2}^{2}.$$

In the following, we show that this change leads to estimation everywhere in k-space, not just indices in ${\left(\Lambda \cap \Omega \right)}^{c}$.

**Claim 1.**  *Consider the random variables $Y=M_{\Omega }\left(Y_{0}+N\right)$ and $\tilde{Y}=M_{\Lambda \cap \Omega }\left(Y+\tilde{N}\right)$, where N and $\tilde{N}$ are zero-mean Gaussians distributed with variances of $\sigma_{n}^{2}$ and $\alpha^{2}\sigma_{n}^{2}$, respectively. When Equations (9) and (10) hold, minimizing*

(27) $$\begin{array}{c} \theta *=\underset{\theta }{\arg\min}\;E[∥M_{\Omega }\left(f_{\theta }\left(\tilde{Y}\right)-Y\right){∥}_{2}^{2}\left|\tilde{Y}\right] \end{array}$$

*yields a network with parameters that satisfies*

(28) $$\begin{array}{c} f_{\theta *}\left(\tilde{Y}\right)=E\left[Y_{0}+N|\tilde{Y}\right]. \end{array}$$

**Proof.**  See Appendix A. □

The differences between Equation (27) and the standard SSDU loss Equation (11) are the change from $M_{\Omega \setminus \Lambda }$ to $M_{\Omega }$ and the inclusion of noise in the data, *Y*. Intuitively, since $M_{\Omega }=M_{\Omega \setminus \Lambda }+M_{\Lambda \cap \Omega }$, the mask change extends Equation (11) to include entries in $M_{\Lambda \cap \Omega }$. Therefore, upon inference, the network learns to map to entries in $M_{{\left(\Lambda \cap \Omega \right)}^{c}}$, as stated in Result 2, *and* $M_{\Lambda \cap \Omega }$, which comes from the additional indices in the loss. In other words, it learns to map to everywhere in k-space. The inclusion of noise in the target simply implies that the network will learn to map to the noisy $Y_{0}+N$, as in Equation (15).

Upon inference, we can use a similar approach to Section 3.1, applying the additive Noisier2Noise correction on indices sampled in $\tilde{Y}$. Since the indices sampled in $\tilde{Y}$ are Λ∩Ω, the clean image $Y_{0}$ is estimable with

(29) $$E\left[Y_{0}|\tilde{Y}\right]=M_{\Lambda \cap \Omega }\left(\frac{\left(1+\alpha^{2}\right)f_{\theta *}\left(\tilde{Y}\right)-\tilde{Y}}{\alpha^{2}}\right)+M_{{\left(\Lambda \cap \Omega \right)}^{c}}f_{\theta *}\left(\tilde{Y}\right).$$

Roughly speaking, Robust SSDU can be thought of as a generalization of Noisier2Full to sub-sampled training data. Specifically, Robust SSDU is mathematically equivalent to Noisier2Full when Ω={1,2,…,q} and there is a change in notation of Λ→Ω. More broadly, Robust SSDU can be interpreted as the simultaneous application of additive and multiplicative Noisier2Noise [25,26].

### 3.3. Loss Weighting of Noisier2Full and Robust SSDU

For Noisier2Full and Robust SSDU, the task during training and inference is not identical; during training, the network maps from $\tilde{Y}$ to $Y_{0}+N$ or $M_{\Omega }\left(Y_{0}+N\right)$, while upon inference, it maps from $\tilde{Y}$ to $Y_{0}$ via the α-based correction term. Taking a similar approach to [34,35], this section describes how this can be compensated for by modifying the loss function in such a way that its gradient equals the gradient of the target loss in a conditional expectation.

**Claim 2.**  *Consider the random variables $Y=M_{\Omega }\left(Y_{0}+N\right)$ and $\tilde{Y}=Y+M_{\Omega }\tilde{N}$, where N and $\tilde{N}$ are zero-mean Gaussians distributed with variances of $\sigma_{n}^{2}$ and $\alpha^{2}\sigma_{n}^{2}$, respectively. We define*

(30) $$\begin{array}{c} {\hat{Y}}_{Nr2F}=M_{\Omega }\left(\frac{\left(1+\alpha^{2}\right)f_{\theta }\left(\tilde{Y}\right)-\tilde{Y}}{\alpha^{2}}\right)+M_{\Omega^{c}}f_{\theta }\left(\tilde{Y}\right) \end{array}$$

*where $f_{\theta }$ is an arbitrary function. Then,*

(31) $$\nabla_{\theta }E\left[{∥{\hat{Y}}_{Nr2F}-Y_{0}∥}_{2}^{2}|\tilde{Y}\right]=\nabla_{\theta }E\left[{∥W_{\Omega }\left(f_{\theta }\left(\tilde{Y}\right)-Y_{0}-N\right)∥}_{2}^{2}|\tilde{Y}\right].$$

*where*

(32) $$W_{\Omega }=\frac{1+\alpha^{2}}{\alpha^{2}}M_{\Omega }+M_{\Omega^{c}}.$$

**Proof.**  See Appendix B. □

We therefore suggest replacing the Noisier2Full loss stated in Equation (20) with the right-hand side of Equation (31), which increases the weight of the indices in Ω. Intuitively, it uses the ratio of noise removed during training, which has the variance $\mathrm{Var}\left(\tilde{N}\right)=\alpha^{2}\sigma_{n}^{2}$, and the noise removed upon inference, which has the variance $\mathrm{Var}\left(N+\tilde{N}\right)=\left(1+\alpha^{2}\right)\sigma_{n}^{2}$, to compensate for the difference between the task during training and inference. The following result concerns the analogous expression for Robust SSDU.

**Claim 3.**  *Consider the random variables $Y=M_{\Omega }\left(Y_{0}+N\right)$ and $\tilde{Y}=M_{\Lambda \cap \Omega }\left(Y+\tilde{N}\right)$, where N and $\tilde{N}$ are zero-mean Gaussians distributed with variances of $\sigma_{n}^{2}$ and $\alpha^{2}\sigma_{n}^{2}$, respectively. We define*

(33) $${\hat{Y}}_{RSSDU}=M_{\Lambda \cap \Omega }\left(\frac{\left(1+\alpha^{2}\right)f_{\theta }\left(\tilde{Y}\right)-\tilde{Y}}{\alpha^{2}}\right)+M_{{\left(\Lambda \cap \Omega \right)}^{c}}f_{\theta *}\left(\tilde{Y}\right)$$

*where $f_{\theta }$ is an arbitrary function. Then,*

(34) $$E\left[{∥{\hat{Y}}_{RSSDU}-Y_{0}∥}_{2}^{2}|\tilde{Y}\right]=\nabla_{\theta }E\left[{∥W_{\Omega ,\Lambda }M_{\Omega }\left(f_{\theta }\left(\tilde{Y}\right)-Y\right)∥}_{2}^{2}|\tilde{Y}\right]$$

*where*

(35) $$W_{\Omega ,\Lambda }=\frac{1+\alpha^{2}}{\alpha^{2}}M_{\Lambda \cap \Omega }+P^{\frac{1}{2}}M_{\Omega \setminus \Lambda }$$

*and $P=E{\left[M_{\Omega \setminus \Lambda }\right]}^{-1}E\left[M_{{\left(\Lambda \cap \Omega \right)}^{c}}\right]$.*

**Proof.**  See Appendix C. □

The $M_{\Lambda \cap \Omega }$ coefficient has a similar role to the $M_{\Omega }$ coefficient in Equation (31). The $M_{\Omega \setminus \Lambda }$ coefficient compensates for the variable density of Ω and Λ and was first proposed in [25], where it was shown to improve the reconstruction quality and robustness of the distribution of Λ for standard SSDU without denoising (where [25] uses ${\left(1-K\right)}^{-1}$, this paper uses the more compact P).

The weightings can be thought of as entry-wise modifications of the learning rate [25]. Neither weighting matrices change θ*, so the proofs of Noisier2Full and Robust SSDU from Section 3.1 and Section 3.2 hold. Rather, the role of the weights is to improve the finite-sample case in practice, where θ* is estimated with $\hat{\theta }$; see Section 5 for an empirical evaluation. Throughout the remainder of this paper, “Noisier2Full” and “Robust SSDU” refer to the versions with the loss weightings proposed in this section and versions without such weightings are explicitly referred to as “Unweighted Noisier2Full” and “Unweighted Robust SSDU”.

## 4. Materials and Methods

### 4.1. Description of Data

We primarily used the multi-coil brain data from the publicly available fastMRI dataset [36] (available from https://fastmri.med.nyu.edu, accessed on 1 October 2021). We only used data that had 16 coils so that the training, validation, and test sets contained 2004, 320, and 224 slices, respectively. The slices were normalized so that the cropped RSS estimate had a maximum of 1. Here, the cropped RSS was defined as $Z((\sum_{c}^{N_{c}}|F^{H}y_{c}{|}^{2}{)}^{\frac{1}{2}})$, where the subscript *c* refers to all entries on the *c*th coil, $F^{H}$ is the conjugate transpose of the discrete Fourier transform, $N_{c}$ is the number of coils, and *Z* is an operator that crops to a central 320×320 region. RSS images were used for normalization and visualization only; otherwise, the raw, complex, multi-coil, k-space data were used. We retrospectively sub-sampled column-wise with the central 10 lines fully sampled and and the others randomly drawn with polynomial density, with the probability density scaled to achieve a desired acceleration factor, $R_{\Omega }=q/\sum_{j}p_{j}$. For $R_{\Omega }=4$ and $\sigma_{n}=0.04$, we also trained the methods on 2D Bernoulli sampling, where the sampling was random and independent, and also with polynomial variable density. For each case, the distribution of $M_{\Lambda }$ was the same type as the first [25]. The data were treated as noise-free, and we generated white, complex Gaussian measurement noise with the standard deviation $\sigma_{n}$ to simulate noisy conditions.

We also tested the methods’ performance on the 0.3T dataset M4Raw [37]. For this dataset, which prospectively had a low SNR, no further noise was added. Rather, the noise covariance matrix was estimated using the fully sampled image via a 30×30 square of background from each corner and the data were whitened by left-multiplying with the inverse covariance matrix so that all data had a noise standard deviation of 1. The same column-wise sub-sampling was used as described above for the fastMRI data. Although it was more realistic for the simulated noise setting of fastMRI, for M4Raw we had no “ground truth”, so it was only possible to evaluate the methods’ performance qualitatively.

An implementation of our method in PyTorch is available on GitHub (https://github.com/charlesmillard/robust_ssdu, accessed on 7 December 2023).

### 4.2. Comment on Proposed Methods in Practice

The theoretical guarantees for Noisier2Full and Robust SSDU use the further noisy, possibly further sub-sampled ${\tilde{y}}_{s}$ as the input to the network upon inference. In practice, as suggested in the original Noisier2Noise [26] and SSDU [13] papers, we used $y_{s}$ as the input to the network upon inference, so that the estimate

(36) $${\hat{y}}_{s}=M_{\Omega_{s}}\left(\frac{\left(1+\alpha^{2}\right)f_{\hat{\theta }}\left(y_{s}\right)-y_{s}}{\alpha^{2}}\right)+M_{\Omega_{s}^{c}}f_{\hat{\theta }}\left(y_{s}\right)$$

was used in place of Equations (24) and (29). Although this deviates from strict theory, and is not guaranteed to be correct in a conditional expectation, we found that it achieves better reconstruction performance in practice; see [25,26] for a detailed empirical evaluation. All subsequent results for the proposed methods use this estimate upon inference.

### 4.3. Comparative Training Methods

The training methods evaluated in this paper are summarized in Table 1.

For the noise-free, fully sampled training data, fully supervised training could be employed, where the loss was computed between the output of the network $f_{\theta }\left(y_{t}\right)$ and the noise-free, fully sampled target $y_{0,t}$; see Table 1. Although it was possible in principle to have higher-SNR data during training than upon inference by acquiring multiple averages [37], such datasets would require an extended acquisition time and are rare in practice. Nonetheless, training a network on this type of data via simulation is instructive as a best-case target. This method is referred to as the “fully supervised benchmark” throughout this paper.

For the noisy, fully sampled training data, we employed three training methods: Unweighted Noisier2Full, Noisier2Full and the standard approach Supervised w/o denoising, as described in Section 3. We did not compare them to Noise2Inverse [38] as it was designed for learned, denoising but fixed reconstruction operators.

For the more challenging scenario where noisy, sub-sampled training data were available, we compared Robust SSDU to the original version of SSDU, which reconstructs sub-sampled data but does not denoise. We refer to this as “Standard SSDU”. To our knowledge, the only existing training method that explicitly aims to simultaneously remove noise and reconstruct incoherently sampled data in a fully self-supervised manner is Noise2Recon-SS [27], which, like Robust SSDU, includes adding further noise to the sub-sampled data. However, Noise2Recon-SS has a number of key differences to the method proposed in this paper. With an $\ell_{2}$ k-space loss, training with Noise2Recon-SS consists of minimizing

(37) $$\hat{\theta }=\underset{\theta }{\arg\min}\sum_{t\in T}∥M_{\Omega_{t}\setminus \Lambda_{t}}\left(f_{\theta }\left(M_{\Lambda_{t}}y_{t}\right)-y_{t}\right){∥}_{2}^{2}+\lambda {∥f_{\theta }\left(y_{t}+M_{\Omega_{t}}{\tilde{n}}_{t}\right)-f_{\theta }\left(M_{\Lambda_{t}}y_{t}\right)∥}_{2}^{2},$$

where λ is a hand-selected weighting. We used λ=1 throughout, which we found performed reasonably well across the range of sampling patterns, noise levels, and datasets explored. We note that it may be possible in principle to improve Noise2Recon-SS’s performance by tuning λ to specific datasets, reconstruction patterns, and noise levels. However, since no ground truth is available, such tuning cannot be performed quantitatively, so fixing λ for all experiments is a reasonable reflection of the method’s performance in practice. The $\ell_{2}$ loss in k-space was used so that it could be fairly compared to the other methods in this paper, but we note that [27] used image-domain losses. The first term was based on SSDU, and the second ensured that $f_{\theta }\left(y_{t}+M_{\Omega_{t}}{\tilde{n}}_{t}\right)$ and $f_{\theta }\left(M_{\Lambda_{t}}y_{t}\right)$ yielded similar outputs so that the method was in a sense robust to ${\tilde{n}}_{t}$. Upon inference, Noise2Recon-SS uses ${\hat{y}}_{s}=f_{\hat{\theta }}\left(y_{s}\right)$; there is no correction term. We emphasize that, unlike the proposed Robust SSDU, there is no theoretical evidence that Noise2Recon-SS recovers a clean image as expected.

In [20], an untrained denoising algorithm was appended to a reconstruction network. To test this, we denoised the RSS output of Supervised w/o denoising and Standard SSDU with the popular BM3D algorithm [39], which is designed for Gaussian noise. Although the measurement noise was Gaussian, the reconstruction error of the RSS image was not Gaussian in general [40]. Therefore, unlike the proposed methods, BM3D did not accurately model the noise characteristics [41]. Nonetheless, we found that these methods performed reasonably well in practice.

### 4.4. Network Architecture

For all methods considered in this paper, the function $f_{\theta }$ is defined to be k-space to k-space, but it is otherwise agnostic to the network architecture. Architectures can include inverse Fourier transforms, so convolutional layers may be applied in the image domain. We emphasize that the experiments in this paper are designed to compare the performance of the training *method*, not to provide a comprehensive evaluation of possible architectures, which is a somewhat orthogonal goal.

We employed a network architecture based on the Variational Network (VarNet) [7,42], which is available as part of the fastMRI package [36]. VarNet consists of a coil sensitivity map estimation module followed by a series of “cascades”. The k-space estimate at the *k*th cascade takes the form

(38) $${\hat{y}}_{k+1}={\hat{y}}_{k}-\eta_{k}M_{in}\left({\hat{y}}_{k}-y_{in}\right)+G_{\theta_{k}}\left({\hat{y}}_{k}\right)$$

where $y_{in}$ and $M_{in}$ are the input k-space and sampling mask, respectively, and the *t* or *s* index is dropped for legibility. We use the generic subscript in here because the input is not the same for every method; for instance, the fully supervised training and Noisier2Full have $M_{in}=M_{\Omega_{t}}$ and $M_{in}=M_{\Lambda_{t}\cap \Omega_{t}}$, respectively. Here, $\eta_{k}$ is a trainable parameter and $G_{\theta_{k}}\left({\hat{y}}_{k}\right)$ is a neural network with cascade-dependent parameters, $\theta_{k}$, referred to as a “refinement module”, which was an image-domain U-net [43] with real weights in [7,42].

VarNet was originally constructed for reconstruction only, without explicit denoising. For joint reconstruction and denoising, we propose partitioning $G_{\theta_{k}}\left({\hat{y}}_{k}\right)$ into two functions:

(39) $$\begin{array}{c} G_{\theta_{k}}\left({\hat{y}}_{k}\right)=M_{in}G_{\theta_{k}^{D}}\left({\hat{y}}_{k}\right)+\left(1-M_{in}\right)G_{\theta_{k}^{R}}\left({\hat{y}}_{k}\right). \end{array}$$

**Figure 2:**
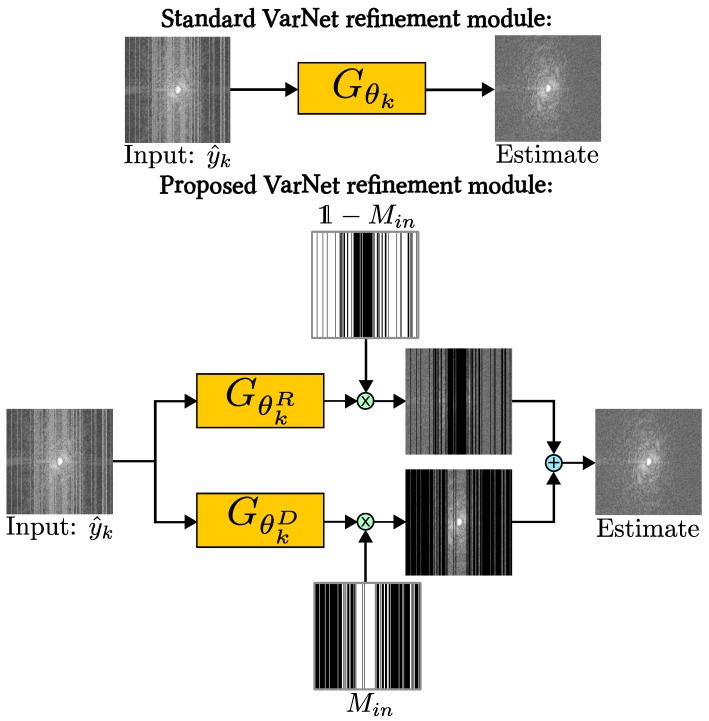
The refinement module for the proposed architecture Denoising VarNet, which trains two networks in parallel, removing noise and aliasing separately.

This refinement module is illustrated in Figure 2. We refer to the architecture with the proposed refinement module as “Denoising VarNet” throughout this paper. We used a U-net [43] for both $G_{\theta_{k}^{D}}\left({\hat{y}}_{k}\right)$ and $G_{\theta_{k}^{R}}\left({\hat{y}}_{k}\right)$, although we note that, in general, these functions need not be the same. We used 5 cascades, giving a network with $2.5\times 10^{7}$ parameters.

### 4.5. Training Details

We used the Adam optimizer [44] and trained for 100 epochs with a learning rate of $10^{-3}$. The $\Omega_{t}$ and $n_{t}$ were fixed but the $\Lambda_{t}$ and ${\tilde{n}}_{t}$ were re-generated once per epoch [45], which we found considerably reduced susceptibility to overfitting. As in [25], we used the same distribution of $\Lambda_{t}$ as $\Omega_{t}$ but with parameters selected to give a sub-sampling factor of $R_{\Lambda }=q/\sum_{j}{\tilde{p}}_{j}=2$ unless otherwise stated. The choice of α is discussed in Section 5.2. Unless otherwise stated, the training methods were evaluated on data generated with $\sigma_{n}\in \left\{0.02,0.04,0.06,0.08\right\}$ and $R_{\Omega }\in \left\{4,8\right\}$. We note that the noise’s standard deviation, not the SNR, was fixed and that for each training method, $\sigma_{n}$ and $R_{\Omega }$, we trained a separate network from scratch.

### 4.6. Performance Metrics

Since each of the methods were trained using a squared error loss in k-space, we primarily focused on the k-space normalized mean squared error (NMSE) over the test set, defined as $\frac{1}{\left|S\right|}\sum_{s\in S}∥{\hat{y}}_{s}-y_{0,s}{∥}_{2}^{2}/{∥y_{0,s}∥}_{2}^{2}$ where ${\hat{y}}_{s}$ is an estimate of k-space. Since the score was in k-space, it was not possible to compute the NMSE of methods that employed BM3D, which acted on the magnitude image so did not retain the complex phase. The peak signal-to-noise ratio (PSNR) was also computed but was found to display very similar trends to the NMSE so is not shown for brevity.

We also computed the mean structural similarity (SSIM) [46] on the RSS images. We emphasize that the networks were *not* trained to minimize the SSIM directly, so such scores are somewhat incidental to the primary NMSE results and not necessarily fundamental to the method.

## 5. Results

### 5.1. Evaluation of Denoising VarNet

**Figure 3:**
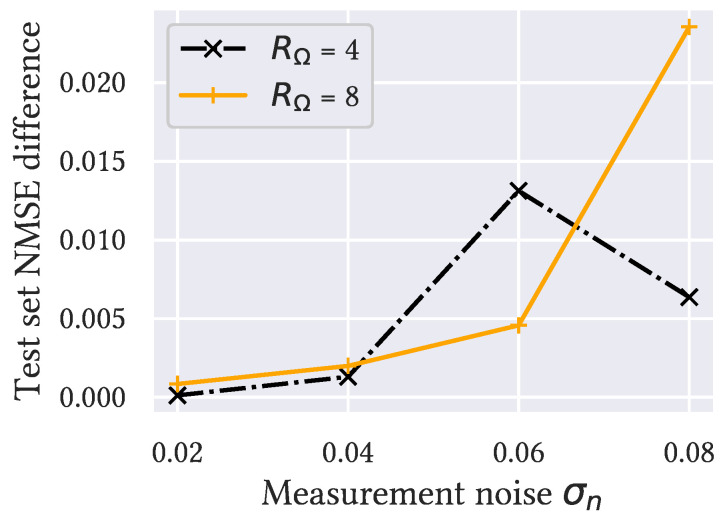
The difference between the test set loss of Standard VarNet and the proposed Denoising VarNet for the benchmark training method. All differences are positive, showing that Denoising VarNet outperformed Standard VarNet, especially for a large $\sigma_{n}$.

To evaluate the performance of the proposed Denoising VarNet architecture, we trained the best-case baseline for Standard VarNet with ten cascades and that for Denoising VarNet with five cascades so that they had roughly the same number of parameters. Figure 3 shows that Denoising VarNet outperformed Standard VarNet on the test set for all considered $R_{\Omega }$ and $\sigma_{n}$, especially for more challenging acceleration factors and noise levels.

### 5.2. Robustness to α

**Figure 4:**
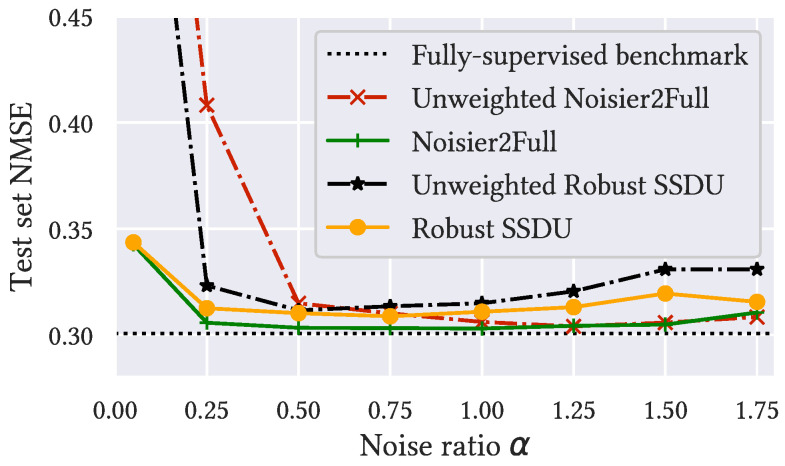
The robustness to α of Noisier2Full, Robust SSDU, and their weighted versions at $R_{\Omega }=8$ and $\sigma_{n}=0.06$. The performance of the fully supervised benchmark, which did not depend on α, is also shown. The weighted versions were substantially more robust, especially for small α: at α=0.05, the values of the Unweighted Noisier2Full and Robust SSDU, which are excluded from the visualization, were 0.70 and 0.62, respectively.

To evaluate the robustness to α, we trained Noisier2Full, Robust SSDU, and their weighted variants for α∈{0.05,0.25,0.5,0.75,1,1.25,1.5,1.75}. We focused solely on the case where $R_{\Omega }=8$ and $\sigma_{n}=0.06$. The performance with the test set is shown in Figure 4, which shows that the weighted versions were considerably more robust. The weighted and unweighted minima were at α=1 and 1.25 for Noisier2Full and α=0.75 and 0.5 for Robust SSDU, respectively. We employed these values of α for all experiments in Section 5.3 and Section 5.4; we assumed that the tuned α at $R_{\Omega }=8$ and $\sigma_{n}=0.06$ was a reasonable approximation of the optimum for every evaluated $R_{\Omega }$ and $\sigma_{n}$.

### 5.3. Task A: Fully Sampled, Noisy Training Data

**Figure 5:**
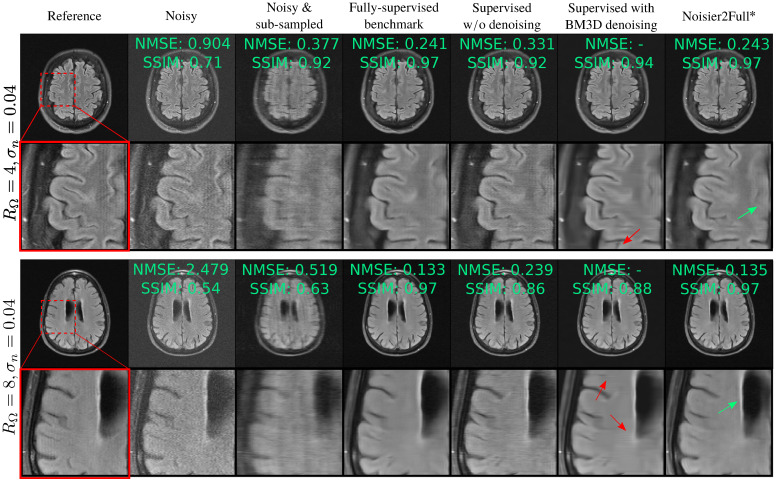
Reconstructions when fully sampled, noisy data are available for training. “Noisy” and “Noisy and sub-sampled” refer to the RSS reconstruction of $y_{0,s}+n_{s}$ and $M_{\Omega_{s}}\left(y_{0,s}+n_{s}\right)$, respectively. While there is clear noise in Supervised w/o denoising’s reconstruction, the proposed method, which is indicated with an asterisk, performs very similarly to the fully supervised benchmark. The red arrows show artifacts for Supervised with BM3D, and the green arrows show the improved recovery and contrast of fine features for Noisier2Full.

Rows 3–5 of Table 2 show how the test set’s NMSE of networks trained on fully sampled, noisy data compares to the fully supervised benchmark. Supervised w/o denoising’s performance significantly degraded as $\sigma_{n}$ increased; for $R_{\Omega }=8$ and $\sigma_{n}=0.08$, Supervised w/o denoising’s test set loss was approximately double that of the fully supervised benchmark. In contrast, Noisier2Full consistently performed similarly to the benchmark; its NMSE was within 0.008 for all $\sigma_{n}$ and $R_{\Omega }$. The performance of Unweighted Noisier2Full was slightly poorer than the weighted version, especially for high noise levels for the more challenging acceleration factor of $R_{\Omega }=8$. Two reconstruction examples are shown in Figure 5. Here, and throughout this paper, the example reconstructions show the image-domain RSS cropped to a central 320×320 region. The k-space’s NMSE and SSIM are also shown. Appendix D shows the mean SSIM for the test set for all methods.

### 5.4. Task B: Sub-Sampled, Noisy Training Data

Rows 6–9 of Table 2 show the test set’s loss for the methods designed for sub-sampled, noisy training data. Robust SSDU performed within 0.012 of the fully supervised benchmark, despite only having access to noisy, sub-sampled training data. Noise2Recon-SS performed well in some cases, particularly at $R_{\Omega }=4$, but was consistently outperformed by both variants of Robust SSDU. To determine whether the observed NMSE improvements in Robust SSDU were statistically significant, a one-sided Wilcoxon signed-rank test was performed with a *p*-value of 0.01. It was found that the NMSE differences in both versions of Robust SSDU compared to Standard SSDU and Noise2Recon-SS were indeed statistically significant for both acceleration factors and all noise levels. Differences between Unweighted Robust SSDU and Robust SSDU were not significant except for the case where $R_{\Omega }=4$ and $\sigma_{n}=0.06$.

**Figure 6:**
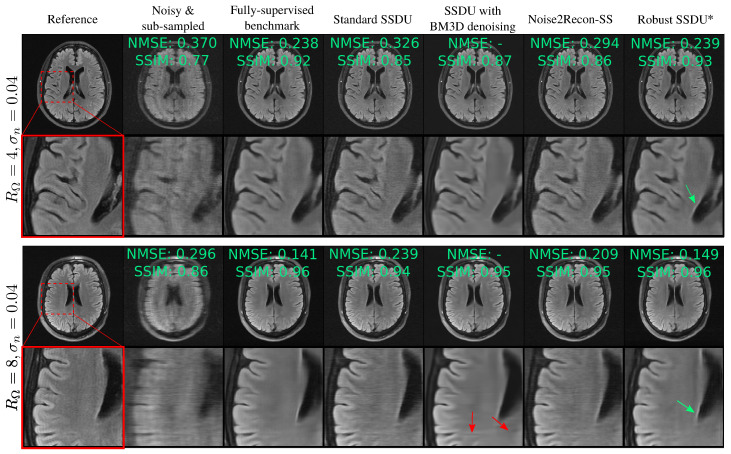
Example reconstructions for networks trained on noisy, sub-sampled data. The proposed method, Robust SSDU, highlighted with an asterisk, performed very similarly to the fully supervised benchmark, even at $R_{\Omega }=8$. Red arrows highlight hallucinated features in the SSDU with BM3D image, whereas green arrows highlight good recovery of edge features in the Robust SSDU reconstructions.

**Figure 7:**
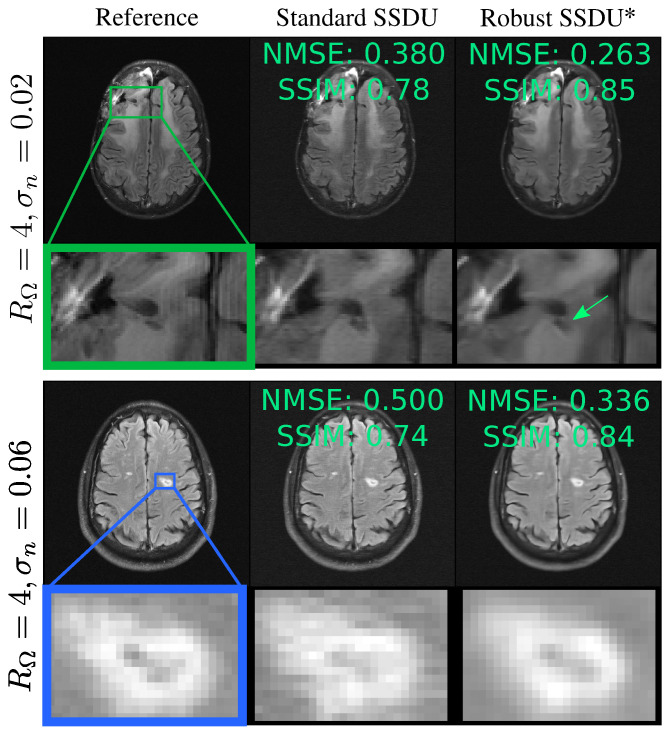
Clinical regions of interest annotated via fastMRI+ [47]. The top image shows a resection cavity and the bottom shows a lacunar infarct. The proposed method, Robust SSDU, highlighted with an asterisk, has improved sharpness compared to Standard SSDU, which has reconstruction errors arising from measurement noise. The arrow highlights improved recovery of infarct geometry.

**Figure 8:**
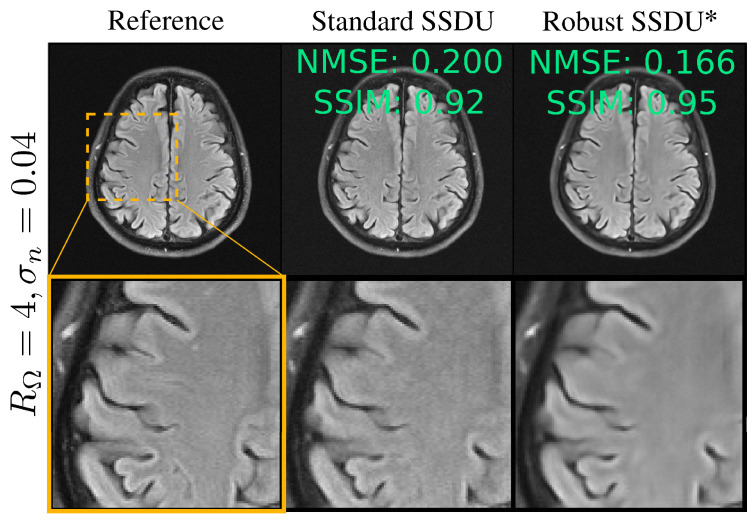
Example reconstruction for 2D Bernoulli sampling. For Standard SSDU, the test set’s NMSE and SSIM were 0.383 and 0.72, respectively, and for Robust SSDU, highlighted with an asterisk, the test set’s NMSE and SSIM were 0.316 and 0.75, respectively.

Figure 6 shows example reconstructions, qualitatively demonstrating similar performance to the fully supervised benchmark. Figure 7 compares Standard SSDU and Robust SSDU using clinical expert bounding boxes from fastMRI+ [47], which shows that the proposed method had substantially enhanced pathology visualization. For the 2D Bernoulli sampling, we found that a lower $R_{\Lambda }$ and α achieved better performance in practice; we used $R_{\Lambda }=1.5$ and α=0.5. Figure 8 compares Standard SSDU and Robust SSDU for the 2D Bernoulli sampled data at $R_{\Omega }=4$ and $\sigma_{n}=0.04$, showing that the denoising effect was not specific to column-wise sampling.

## 6. Discussion

Figure 3 shows that the proposed Denoising VarNet consistently outperformed the Standard VarNet architecture. We understand this to be a consequence of the difference between the distributions of errors due to sub-sampling or measurement noise; Standard VarNet removed both contributions to the error in a single U-net per cascade, while Denoising VarNet simplified the task by decomposing the contributions to the error so that each of the two U-nets per cascade were specialized for the two distinct error distributions.

The improvement in robustness for the weighted versions, shown in Figure 4, was especially prominent for a small α. For instance, at α=0.05, the unweighted variant of Noisier2Full was 0.50 from the benchmark, while the weighted variant was only 0.04 away. For a large α, the α-based weighting was closer to 1, so the weighted Noisier2Full tended to the unweighted method and the difference in performance was small. For instance, when α=1.75, the α-based weighting was 1.33, so it had a relatively marginal effect. Although the performances of the methods were reasonably similar for a tuned α, we recommend using the weighted version in practice due to its improved robustness to α. We emphasize that α tuning was only possible here because the noise and sub-sampling were simulated retrospectively; if the data were prospectively noisy and sub-sampled, it would not possible to evaluate the fidelity of the estimate and the ground truth. Robustness to hyperparameters such as α is therefore of great importance for the method’s usefulness in practice.

The examples in Figure 5, Figure 6 and Figure 7 show that proposed methods are qualitatively very similar to the fully supervised benchmark and substantially improve over methods without denoising, whose reconstructions are visibly corrupted with measurement noise. The examples exhibited some loss of detail and blurring at tissue boundaries, especially at $R_{\Omega }=8$. However, the extent of detail loss was similar in the benchmark, indicating that the loss of detail was not a limitation of the proposed methods. Rather, the qualitative performance was limited by other factors such as the architecture, dataset, and choice of loss function. This can also be explained in part by noting that the high-frequency regions of k-space, which provide fine details, typically have a smaller signal so are particularly challenging to recover in the presence of significant measurement noise.

Table 2 shows that the NMSE of the noisy, sub-sampled input to the network was *lower* for the higher acceleration factor. This counter-intuitive incidental finding can be understood by noting that the spectral density was typically highly concentrated towards the center, so much of the k-space had a small magnitude. Therefore, even for moderate noise levels, zero may have been closer to the ground truth than the noisy data, so masking out such regions may have improved the NMSE. This was also reflected in the NMSE scores of the reconstructed images. However, we note that this effect was not generally reflected in the qualitative performance of the methods, which we found more frequently exhibited oversmoothing and artifacts for higher acceleration. We believe this to be because the masked data were biased, so it was more difficult to achieve a high-quality qualitative performance in practice.

The pseudo-denoising effect described in Section 3 is visible in Figure 5, showing less noise in Supervised w/o denoising than Noisy. Table 2 shows that Standard SSDU performs very similarly to Supervised w/o denoising quantitatively and exhibits a similar pseudo-denoising effect in Figure 6.

Although Noise2Recon-SS improved over Standard SSDU, there was a substantial difference between its performance and that of the proposed Robust SSDU both qualitatively and quantitatively. In [27], Noise2Recon-SS was not compared to a fully supervised benchmark; it was only shown to have improved performance compared to Standard SSDU, consistent with the results here. The experimental evaluation in [27] focused on robustness to out of distribution (OOD) shifts, where the training and inference measurement noise variances were not necessarily the same. Another difference was that Noise2Recon-SS’s simulated noise in [27] had a standard deviation randomly selected from a fixed range, while the experiments here fixed the simulated noise’s standard deviation so that it could be properly compared to the proposed methods.

Robust SSDU required only a few additional cheap computational steps compared to standard training: the addition or multiplication of the further noise and sub-sampling mask, respectively, and the α-based correction upon inference. Accordingly, the compute time and memory requirements of the proposed methods were found to be very similar to those of Supervised w/o denoising or Standard SSDU. In contrast, Noise2Recon-SS used both $M_{\Lambda_{t}}y_{t}$ and $y_{t}+M_{\Omega_{t}}{\tilde{n}}_{t}$ as the network inputs during training so required twice as many forward passes to train the network compared to Robust SSDU. Accordingly, we found that Noise2Recon-SS required approximately twice as much memory as and took around two times longer per epoch than the proposed methods.

**Figure 9:**
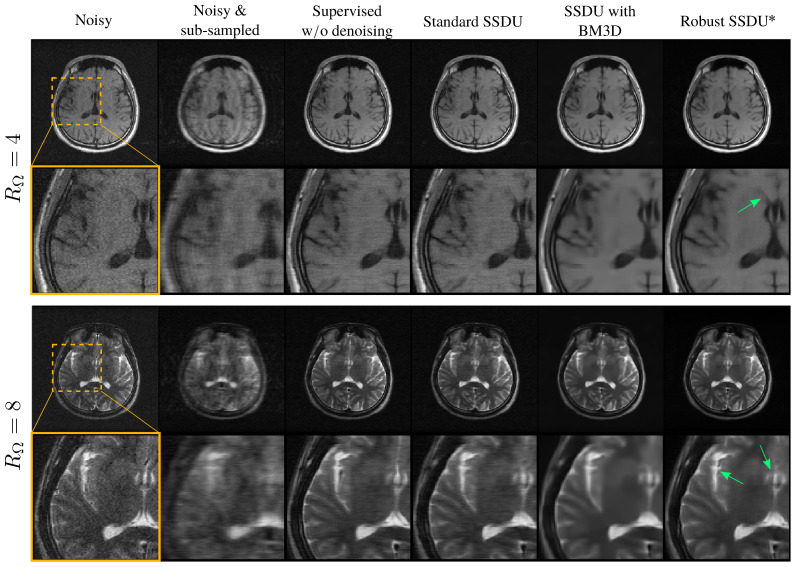
The qualitative performance of the proposed method with the prospectively noisy, low-field dataset M4Raw. While SSDU with BM3D and Robust SSDU (highlighted with an asterisk) both demonstrate a denoising effect, Robust SSDU exhibits improved contrast and visibly sharper boundaries, highlighted by the green arrows.

In general, Supervised with BM3D and SSDU with BM3D both performed well qualitatively. We also found that in many cases these methods had an mean SSIM that exceeded even the fully supervised benchmark; see Appendix D for a detailed discussion. However, for some images, such as those shown with the red arrows in Figure 5 and Figure 6, these methods generated potentially clinically misleading artifacts. We believe this to be a consequence of the mismatch between its Gaussian noise model and the actual error of the RSS estimate, which could lead to unreliable noise removal, especially at a high $R_{\Omega }$. We also found that SSDU with BM3D often led to more oversmoothing and less crisp tissue boundaries than Robust SSDU, which is particularly prominent in the M4Raw examples of Figure 9. Another disadvantage was the computational expense of the BM3D algorithm; we found that the reconstruction time of SSDU with BM3D was around 100 times longer per slice than that of Robust SSDU upon inference.

Another existing method designed for noisy, sub-sampled training data is the robust equivariant imaging (REI) method [48,49]. We did not compare REI as it was designed for reconstruction tasks with a fixed sampling pattern: the $\Omega_{t}$ is the same for all *t*. This sampling set assumption is central to its use of equivariance and contrasts with the methods proposed here, which assumed that the sampling mask was an instance of a random variable that satisfied $p_{j}>0$ everywhere. However, REI’s suggestion to use Stein’s unbiased risk estimate (SURE) [50] to remove measurement noise would be feasible in combination with SSDU and warrants further investigation in future work.

The theoretical work presented in this paper only applies to the case of $\ell_{2}$ minimization, which can lead to blurry reconstructions. However, it has been established that Standard SSDU can be applied with other losses such as an entry-wise mixed $\ell_{1}$-$\ell_{2}$ loss in k-space [13]. We found that Robust SSDU with an $\ell_{2}$ loss with Λ∩Ω and mixed $\ell_{1}$-$\ell_{2}$ loss with Ω∖Λ also performed competitively with a suitable benchmark in practice (results are not shown for brevity). Future work includes establishing whether Robust SSDU can be modified to be applicable to other loss functions, including potential losses in the RSS image.

The methods presented here also assumed that the distribution of $M_{\Omega }$ was fixed; a modification of the method for dealing with a range of sub-sampling patterns and acceleration factors is a potential avenue for future work. It would also be desirable to develop an approach that automatically tunes α and the distribution of $M_{\Lambda }$, whose optimal values are specific to the noise model, $M_{\Omega }$ distribution, and dataset.

The additive Noisier2Noise was designed for Gaussian noise; the α-based correction term applied upon inference is derived on the assumption that the noise is Gaussian [26]. Therefore, the naive application of Robust SSDU would not be expected to perform well for other measurement noise distributions. Future work includes extending the framework to other distributions and sources of error such as other system noise or physiological motion, which has a more complex distribution that may itself be learned [51,52].

**Figure 10:**
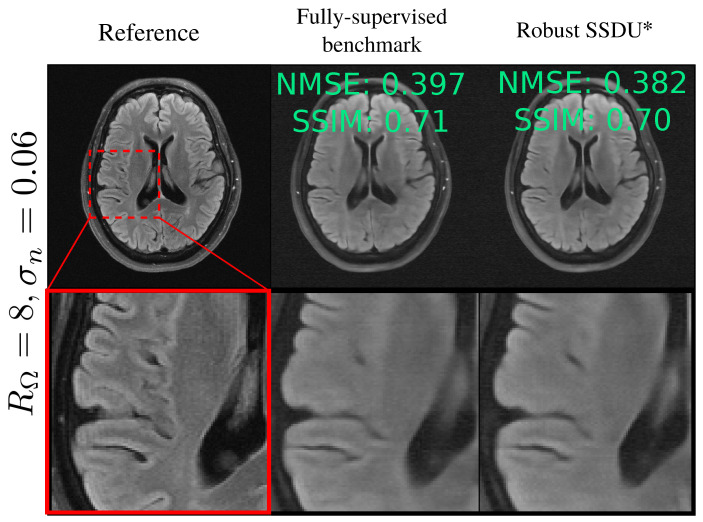
Poor recovery of fine details for ambitious sub-sampling and noise levels at $R_{\Omega }=8$ and $\sigma_{n}=0.08$ for both the Fully-supervised benchmark, and Robust SSDU (highlighted with an asterisk).

Although Denoising VarNet was found to offer improved performance compared to Standard VarNet, the evaluation of possible architectures for simultaneous denoising and reconstruction was not extensive in this paper and warrants future work. For instance, simpler models or the combination of multiple models in parallel [53] may improve computational expense or reconstruction quality in practice. Improvements to the network architecture would be necessary for the more ambitious sub-sampling factors and noise levels investigated in this paper. For instance, at $R_{\Omega }=8$ and $\sigma_{n}=0.08$, Robust SSDU and the fully supervised benchmark both displayed a significant loss of details at tissue boundaries and were of insufficient quality for clinical applications; see Figure 10.

## Figures and Tables

**Table 1:** The training methods evaluated in this paper, where $y_{t}=M_{\Omega_{t}}\left(y_{0,t}+n_{t}\right)$ and the asterisk denotes the proposed methods. Here, and throughout this paper, the subscripts *t* and *s* index the training and test sets, respectively. The function BM3D(·) is defined here to include an RSS transform so that the denoiser acts on the RSS image. The double lines are used to separate types of data available for training. The unweighted variants of Noisier2Full and Robust SSDU, which are not stated here for brevity, are equivalent to the weighted versions with $W_{\Omega_{t}}=1$ and $W_{\Omega_{t},\Lambda_{t}}=1$.

Name	Training Data	Loss	Estimate upon Inference
Fully supervised benchmark	$y_{0,t}$	$\sum_{t\in T}{∥f_{\theta }\left(y_{t}\right)-y_{0,t}∥}_{2}^{2}$	$f_{\theta }\left(y_{s}\right)$
Supervised w/o denoising	$y_{0,t}+n_{t}$	$\sum_{t\in T}{∥f_{\theta }\left(y_{t}\right)-\left(y_{0,t}+n_{t}\right)∥}_{2}^{2}$	$f_{\hat{\theta }}\left(y_{s}\right)$
Supervised with BM3D denoising	$y_{0,t}+n_{t}$	$\sum_{t\in T}{∥f_{\theta }\left(y_{t}\right)-\left(y_{0,t}+n_{t}\right)∥}_{2}^{2}$	BM3D$\left(f_{\hat{\theta }}\left(y_{s}\right)\right)$
Noisier2Full *	$y_{0,t}+n_{t}$	$\sum_{t\in T}{∥W_{\Omega_{t}}\left(f_{\theta }\left(y_{t}+M_{\Omega_{t}}{\tilde{n}}_{t}\right)-\left(y_{0,t}+n_{t}\right)\right)∥}_{2}^{2}$	$M_{\Omega_{s}}\left(\frac{\left(1+\alpha^{2}\right)f_{\hat{\theta }}\left(y_{s}\right)-y_{s}}{\alpha^{2}}\right)+M_{\Omega_{s}^{c}}f_{\hat{\theta }}\left(y_{s}\right)$
Standard SSDU	$y_{t}$	$\sum_{t\in T}{∥M_{\Omega_{t}\setminus \Lambda_{t}}\left(f_{\theta }\left(M_{\Lambda_{t}}y_{t}\right)-y_{t}\right)∥}_{2}^{2}$	$f_{\hat{\theta }}\left(y_{s}\right)$
SSDU with BM3D	$y_{t}$	$\sum_{t\in T}{∥M_{\Omega_{t}\setminus \Lambda_{t}}\left(f_{\theta }\left(M_{\Lambda_{t}}y_{t}\right)-y_{t}\right)∥}_{2}^{2}$	BM3D$\left(f_{\hat{\theta }}\left(y_{s}\right)\right)$
Noise2Recon-SS	$y_{t}$	$\sum_{t\in T}{∥M_{\Omega_{t}\setminus \Lambda_{t}}\left(f_{\theta }\left(M_{\Lambda_{t}}y_{t}\right)-y_{t}\right)∥}_{2}^{2}$ $+\lambda ∥f_{\theta }\left(y_{t}+M_{\Omega_{t}}{\tilde{n}}_{t}\right)-f_{\theta }\left(M_{\Lambda_{t}}y_{t}\right){∥}_{2}^{2}$	$f_{\hat{\theta }}\left(y_{s}\right)$
Robust SSDU *	$y_{t}$	$\sum_{t\in T}{∥W_{\Omega_{t},\Lambda_{t}}M_{\Omega_{t}}\left(f_{\theta }\left(M_{\Lambda_{t}\cap \Omega_{t}}\left(y_{t}+{\tilde{n}}_{t}\right)\right)-y_{t}\right)∥}_{2}^{2}$	$M_{\Omega_{s}}\left(\frac{\left(1+\alpha^{2}\right)f_{\hat{\theta }}\left(y_{s}\right)-y_{s}}{\alpha^{2}}\right)+M_{\Omega_{s}^{c}}f_{\hat{\theta }}\left(y_{s}\right)$

**Table 2:** The methods’ test set’s NMSE with the fastMRI multi-coil brain dataset with standard errors. The double lines separate the type of training data available and bold font is used to denote the best performance within each category. Methods that used BM3D could not be included because the NMSE was computed in k-space and BM3D acted on the magnitude image, so the complex phase was not retained. Table A1 shows a similar table for the SSIM. Asterisks denote proposed methods.

	Acceleration Factor $R_{\Omega }=4$				Acceleration Factor $R_{\Omega }=8$			
	$\sigma_{n}=0.02$	$\sigma_{n}=0.04$	$\sigma_{n}=0.06$	$\sigma_{n}=0.08$	$\sigma_{n}=0.02$	$\sigma_{n}=0.04$	$\sigma_{n}=0.06$	$\sigma_{n}=0.08$
Noisy and sub-sampled	0.210±0.01	0.434±0.01	0.809±0.02	1.333±0.02	0.207±0.02	0.337±0.02	0.554±0.02	0.857±0.02
Fully supervised benchmark	0.167±0.02	0.313±0.02	0.537±0.02	0.850±0.02	0.160±0.02	0.217±0.02	0.301±0.02	0.414±0.02
Supervised w/o denoising	0.187±0.02	0.412±0.02	0.788±0.02	1.314±0.02	0.178±0.02	0.310±0.02	0.527±0.02	0.833±0.02
Unweighted Noisier2Full *	0.170±0.02	0.319±0.02	0.548±0.02	0.870±0.02	0.164±0.02	0.223±0.02	0.315±0.02	0.441±0.02
Noisier2Full *	0.169±0.02	0.312±0.02	0.538±0.02	0.853±0.02	0.162±0.02	0.220±0.02	0.305±0.02	0.422±0.02
Standard SSDU	0.188±0.01	0.413±0.01	0.787±0.01	1.310±0.01	0.180±0.01	0.312±0.01	0.531±0.01	0.838±0.01
Noise2Recon-SS	0.180±0.02	0.377±0.02	0.623±0.02	0.975±0.02	0.173±0.02	0.260±0.02	0.452±0.02	0.691±0.02
Unweighted Robust SSDU *	0.170±0.02	0.314±0.02	0.548±0.02	0.863±0.02	0.162±0.02	0.222±0.02	0.309±0.02	0.424±0.02
Robust SSDU *	0.169±0.02	0.315±0.02	0.543±0.02	0.862±0.02	0.162±0.02	0.224±0.02	0.309±0.02	0.423±0.02

## Data Availability

This paper uses the public dataset fastMRI, available from https://fastmri.med.nyu.edu, accessed on 1 October 2021.

## Appendix A. Proof of SSDU Variant on M Ω

This appendix proves Claim 1 using a similar approach to Appendix B of [25]. Minimization according to Equation (27) yields a network that satisfies

(A1) $$E[M_{\Omega }\left(f_{\theta *}\left(\tilde{Y}\right)-Y\right)|\tilde{Y}]=0$$

We split the conditional expectation into two cases: ${\tilde{Y}}_{j}\neq 0$ and ${\tilde{Y}}_{j}=0$. Throughout this paper, $m_{j}$ and ${\tilde{m}}_{j}$ refer to the *j*th diagonal of $M_{\Omega }$ and $M_{\Lambda }$, respectively.

*Case 1* ($E[m_{j}\left(f_{\theta *}{\left(\tilde{Y}\right)}_{j}-Y_{j}\right)|{\tilde{Y}}_{j}\neq 0]$): When ${\tilde{Y}}_{j}\neq 0$, the measurement model implies that $m_{j}=1$ and $Y_{j}=Y_{0,j}+N_{j}$. Therefore,

(A2) $$E\left[m_{j}\left(f_{\theta *}{\left(\tilde{Y}\right)}_{j}-Y_{j}\right)|{\tilde{Y}}_{j}\neq 0\right]=E\left[f_{\theta *}{\left(\tilde{Y}\right)}_{j}-Y_{0,j}-N_{j}|{\tilde{Y}}_{j}\neq 0\right]$$

*Case 2* ($E[m_{j}\left(f_{\theta *}{\left(\tilde{Y}\right)}_{j}-Y_{j}\right)|{\tilde{Y}}_{j}=0]$): We can use the result derived from Equation (27) in (29) in [25], with $Y_{0,j}$ replaced by $Y_{0,j}+N_{j}$:

(A3) $$E\left[m_{j}\left(f_{\theta *}{\left(\tilde{Y}\right)}_{j}-Y_{j}\right)|{\tilde{Y}}_{j}=0\right]=E\left[f_{\theta *}{\left(\tilde{Y}\right)}_{j}-Y_{0,j}-N_{j}|{\tilde{Y}}_{j}=0\right]\cdot \left(1-k_{j}\right)$$

where

(A4) $$k_{j}=P\left[Y_{j}=0|{\tilde{Y}}_{j}=0\right]=\frac{1-p_{j}}{1-{\tilde{p}}_{j}p_{j}}.$$

*Combining Cases 1 and 2*: Consider the candidate

$$E\left[m_{j}\left(f_{\theta *}{\left(\tilde{Y}\right)}_{j}-Y_{j}\right)|{\tilde{Y}}_{j}\right]=\left\{1-k_{j}\left(1-{\tilde{m}}_{j}m_{j}\right)\right\}E\left[f_{\theta *}{\left(\tilde{Y}\right)}_{j}-Y_{0,j}-N_{j}|{\tilde{Y}}_{j}\right].$$

To verify that this expression is correct, we can check that it is consistent with Cases 1 and 2. For Case 1: if ${\tilde{Y}}_{j}\neq 0$, then ${\tilde{m}}_{j}m_{j}=1$ and the term in curly brackets is 1, so Equation (A5) is consistent with Equation (A2). For Case 2: if ${\tilde{Y}}_{j}=0$, then ${\tilde{m}}_{j}m_{j}=0$ and the term in curly brackets is $1-k_{j}$, so Equation (A5) is consistent with Equation (A3), as required. By Equation (A1),

$$\left\{1-k_{j}\left(1-{\tilde{m}}_{j}m_{j}\right)\right\}E\left[f_{\theta *}{\left(\tilde{Y}\right)}_{j}-Y_{0,j}-N_{j}|{\tilde{Y}}_{j}\right]=0$$

The term in the curly brackets is non-zero for all *j* if $1-k_{j}$ is non-zero for j∉Ω∩Λ, which is true when Equations (9) and (10) hold, where we note that the special case ${\tilde{p}}_{j}=p_{j}=1$ is also allowed since ${\tilde{m}}_{j}m_{j}=1$, always. Given this assumption, dividing by the term in the curly brackets gives the following:

(A5) $$E[f_{\theta *}{\left(\tilde{Y}\right)}_{j}-Y_{0,j}-N_{j}|{\tilde{Y}}_{j}]=0.$$

Vectorizing gives the required result. □

## Appendix B. Proof of Weighted Noisier2Full

To compute the unknown

$$\nabla_{\theta }E\left[{∥{\hat{Y}}_{Nr2F}-Y_{0}∥}_{2}^{2}|\tilde{Y}\right]$$

in terms of the known $Y_{0}+N$, we compute the contributions to the loss in Ω and $\Omega^{c}$ separately, shown in Lemmas A1 and A2, respectively.

**Lemma A1.** 
*Consider the random variables $Y=M_{\Omega }\left(Y_{0}+N\right)$ and $\tilde{Y}=Y+M_{\Omega }\tilde{N}$, where N and $\tilde{N}$ are zero-mean Gaussians distributed with variances of $\sigma_{n}^{2}$ and $\alpha^{2}\sigma_{n}^{2}$, respectively. For an arbitrary function, $f_{\theta }$,*

(A6)
$$\nabla_{\theta }E\left[{∥M_{\Omega }\left({\hat{Y}}_{Nr2F}-Y_{0}\right)∥}_{2}^{2}|\tilde{Y}\right]=\nabla_{\theta }E\left[{∥\frac{1+\alpha^{2}}{\alpha^{2}}M_{\Omega }\left(f_{\theta }\left(\tilde{Y}\right)-Y\right)∥}_{2}^{2}|\tilde{Y}\right].$$

**Proof.** Using $M_{\Omega }\tilde{Y}=M_{\Omega }\left(Y+\tilde{N}\right)$ and $M_{\Omega }Y_{0}=M_{\Omega }\left(Y-N\right)$, the left-hand side of Equation (A6) is

(A7) $$\begin{array}{cc} \; & \nabla_{\theta }E\left[{∥M_{\Omega }\left(\frac{\left(1+\alpha^{2}\right)f_{\theta }\left(\tilde{Y}\right)-\tilde{Y}}{\alpha^{2}}-Y_{0}\right)∥}_{2}^{2}|\tilde{Y}\right]\\ \; & =\nabla_{\theta }E\left[{∥M_{\Omega }\left(\frac{\left(1+\alpha^{2}\right)f_{\theta }\left(\tilde{Y}\right)-Y-\tilde{N}}{\alpha^{2}}-Y+N\right)∥}_{2}^{2}|\tilde{Y}\right]\\ \; & =\nabla_{\theta }E\left[{∥M_{\Omega }\left(\frac{1+\alpha^{2}}{\alpha^{2}}\left(f_{\theta }\left(\tilde{Y}\right)-Y\right)+N-\frac{\tilde{N}}{\alpha^{2}}\right)∥}_{2}^{2}|\tilde{Y}\right]\\ \; & =\nabla_{\theta }E\left[{∥\frac{1+\alpha^{2}}{\alpha^{2}}M_{\Omega }\left(f_{\theta }\left(\tilde{Y}\right)-Y\right)∥}_{2}^{2}|\tilde{Y}\right]+\frac{1+\alpha^{2}}{\alpha^{2}}\nabla_{\theta }E\left[2f_{\theta }{\left(\tilde{Y}\right)}^{H}M_{\Omega }\left(N-\frac{\tilde{N}}{\alpha^{2}}\right)|\tilde{Y}\right] \end{array}$$

where all the terms in the expansion of the $\ell_{2}$ norm in the last step that are not dependent on θ are zeroed by $\nabla_{\theta }$. Now, we show that the second term on the right-hand side of Equation (A7) is zero. Lemma 3.1 from [26] shows that

(A8) $$E\left[M_{\Omega }\tilde{N}|\tilde{Y}\right]=\alpha^{2}E\left[M_{\Omega }N|\tilde{Y}\right],$$

where $M_{\Omega }$ is included as the result only applies to sampled terms. We note that the right-hand side of Equation (A8) scales according to the *variance* of the noise rather than the perhaps more intuitive standard deviation. Following [26], Equation (A8) can be proven by computing the probability $P[N_{j}=n|{\tilde{Y}}_{j},j\in \Omega ]$:

$$\begin{array}{cc} P[N_{j}=n|{\tilde{Y}}_{j},j\in \Omega ] & =P\left[N_{j}=n\right]P\left[{\tilde{N}}_{j}={\tilde{Y}}_{j}-Y_{0,j}-n_{j}\right]\\ \; & \propto \exp\left(-\frac{n^{2}}{2\sigma^{2}}\right)\exp\left(-\frac{{\left({\tilde{Y}}_{j}-Y_{0,j}-n\right)}^{2}}{2\alpha^{2}\sigma^{2}}\right). \end{array}$$

After some algebraic manipulation not shown here for brevity, this distribution can be shown to have the mean $\left({\tilde{Y}}_{j}-Y_{0,j}\right)/\left(1+\alpha^{2}\right)$. A similar computation for ${\tilde{N}}_{j}$ yields a mean of $\alpha^{2}\left({\tilde{Y}}_{j}-Y_{0,j}\right)/\left(1+\alpha^{2}\right)$, giving the *j*th entry of the relationship stated in Equation (A8), conditional on j∈Ω. Since for the alternative j∉Ω both sides are trivially zero, Equation (A8) is correct for all indices. Applying Equation (A8) to the right-hand side of Equation (A7) gives

(A9) $$E\left[f_{\theta }{\left(\tilde{Y}\right)}^{H}M_{\Omega }\left(N-\frac{\tilde{N}}{\alpha^{2}}\right)|\tilde{Y}\right]=f_{\theta }{\left(\tilde{Y}\right)}^{H}E\left[M_{\Omega }\left(N-\frac{\tilde{N}}{\alpha^{2}}\right)|\tilde{Y}\right]=0$$

where the conditional dependence on $\tilde{Y}$ allows the removal of $f_{\theta }\left(\tilde{Y}\right)$ from the expectation. Therefore, the right-hand side of Equation (A7) equals the right-hand side of Equation (A6), as required. □

**Lemma A2.** 
*Consider the random variables Y and $\tilde{Y}$ as defined in Lemma A1. For an arbitrary function $f_{\theta }$,*

(A10)
$$\nabla_{\theta }E\left[{∥M_{\Omega^{c}}\left({\hat{Y}}_{Nr2F}-Y_{0}\right)|\tilde{Y}∥}_{2}^{2}\right]=\nabla_{\theta }E\left[{∥M_{\Omega^{c}}\left(f_{\theta }\left(\tilde{Y}\right)-Y_{0}-N\right)∥}_{2}^{2}|\tilde{Y}\right].$$

**Proof.** Using $M_{\Omega^{c}}M_{\Omega }=0$ and $M_{\Omega^{c}}M_{\Omega^{c}}=M_{\Omega^{c}}$ and the definition of ${\hat{Y}}_{Nr2F}$ in Equation (30), we have $M_{\Omega^{c}}{\hat{Y}}_{Nr2F}=M_{\Omega^{c}}f_{\theta }\left(\tilde{Y}\right)$. Therefore, the left-hand side of Equation (A10) is

(A11) $$\begin{array}{cc} \; & \nabla_{\theta }E\left[{∥M_{\Omega^{c}}\left(f_{\theta }\left(\tilde{Y}\right)-Y_{0}\right)∥}_{2}^{2}|\tilde{Y}\right]\\ \; & =\nabla_{\theta }E\left[{∥M_{\Omega^{c}}\left(f_{\theta }\left(\tilde{Y}\right)-Y_{0}-N+N\right)∥}_{2}^{2}|\tilde{Y}\right]\\ \; & =\nabla_{\theta }E\left[{∥M_{\Omega^{c}}\left(f_{\theta }\left(\tilde{Y}\right)-Y_{0}-N\right)∥}_{2}^{2}+2f_{\theta }{\left(\tilde{Y}\right)}^{H}M_{\Omega^{c}}N|\tilde{Y}\right] \end{array}$$

where, again, all the terms not dependent on θ are zeroed by $\nabla_{\theta }$. The second term is

(A12) $$E\left[f_{\theta }{\left(\tilde{Y}\right)}^{H}M_{\Omega^{c}}N|\tilde{Y}\right]=f_{\theta }{\left(\tilde{Y}\right)}^{H}E\left[M_{\Omega^{c}}N|\tilde{Y}\right]=0$$

where, as in Equation (16), we use the independence of *N* from $\tilde{Y}$ when j∉Ω. Therefore, Equation (A11) equals the right-hand side of Equation (A10), as required. □

To find the $\ell_{2}$ error of ${\hat{Y}}_{Nr2F}$, we use $M_{\Omega }+M_{\Omega^{c}}=1$ and sum the results from Lemmas A1 and A2:

$$\begin{array}{ll} \nabla_{\theta }E\left[{∥{\hat{Y}}_{Nr2F}-Y_{0}∥}_{2}^{2}|\tilde{Y}\right]\; & =\nabla_{\theta }E\left[{∥\left(M_{\Omega }+M_{\Omega^{c}}\right)\left({\hat{Y}}_{Nr2F}-Y_{0}\right)∥}_{2}^{2}|\tilde{Y}\right]\\ & =\nabla_{\theta }E\left[{∥\left(\frac{1+\alpha^{2}}{\alpha^{2}}M_{\Omega }+M_{\Omega^{c}}\right)\left(f_{\theta }\left(\tilde{Y}\right)-Y_{0}-N\right)∥}_{2}^{2}|\tilde{Y}\right] \end{array}$$

as required.

## Appendix C. Proof of Robust SSDU Weighting

Analogous to Appendix B, to compute the unknown

$$\nabla_{\theta }E\left[{∥{\hat{Y}}_{RSSDU}-Y_{0}∥}_{2}^{2}|\tilde{Y}\right]$$

in terms of the known sub-sampled, noisy *Y*, we compute the contributions to the loss from Λ∩Ω and ${\left(\Lambda \cap \Omega \right)}^{c}$ separately. For the contribution from Λ∩Ω, an identical approach to the proof in Lemma A1 can be used with Ω replaced by Λ∩Ω so that

(A13) $$\nabla_{\theta }E\left[{∥M_{\Lambda \cap \Omega }\left({\hat{Y}}_{RSSDU}-Y_{0}\right)∥}_{2}^{2}|\tilde{Y}\right]=\nabla_{\theta }E\left[{∥\frac{1+\alpha^{2}}{\alpha^{2}}M_{\Lambda \cap \Omega }\left(f_{\theta }\left(\tilde{Y}\right)-Y\right)∥}_{2}^{2}|\tilde{Y}\right].$$

The following lemma shows how the remaining loss, which is computed on Ω∖Λ, can be used to estimate the target ground truth loss, which is over ${\left(\Lambda \cap \Omega \right)}^{c}$.

**Lemma A3.** 
*Consider the random variables $Y=M_{\Omega }\left(Y_{0}+N\right)$ and $\tilde{Y}=M_{\Lambda \cap \Omega }\left(Y+\tilde{N}\right)$, where N and $\tilde{N}$ are zero-mean Gaussians distributed with variances of $\sigma_{n}^{2}$ and $\alpha^{2}\sigma_{n}^{2}$, respectively. For an arbitrary function $f_{\theta }$,*

(A14)
$$\nabla_{\theta }E\left[{∥M_{{\left(\Lambda \cap \Omega \right)}^{c}}\left({\hat{Y}}_{RSSDU}-Y_{0}\right)∥}_{2}^{2}|\tilde{Y}\right]=\nabla_{\theta }E\left[{∥P^{1/2}M_{\Omega \setminus \Lambda }\left(f_{\theta }\left(\tilde{Y}\right)-Y\right)∥}_{2}^{2}|\tilde{Y}\right],$$

*where P is defined in Equation (3).*

**Proof.** Since $M_{{\left(\Lambda \cap \Omega \right)}^{c}}{\hat{Y}}_{RSSDU}=M_{{\left(\Lambda \cap \Omega \right)}^{c}}f_{\theta }\left(\tilde{Y}\right)$, the left-hand side of Equation (A14) is

(A15) $$\nabla_{\theta }E\left[{∥M_{{\left(\Lambda \cap \Omega \right)}^{c}}\left(f_{\theta }\left(\tilde{Y}\right)-Y_{0}\right)∥}_{2}^{2}|\tilde{Y}\right]=\nabla_{\theta }E\left[{∥M_{{\left(\Lambda \cap \Omega \right)}^{c}}\left(f_{\theta }\left(\tilde{Y}\right)-Y_{0}-N\right)∥}_{2}^{2}|\tilde{Y}\right],$$

where Lemma A2 with $\Omega^{c}$ replaced by ${\left(\Lambda \cap \Omega \right)}^{c}$ is used. Using ${\left|\cdot \right|}^{2}$ to denote the entry-wise magnitude squared, we can write

(A16) $$\nabla_{\theta }E\left[{∥M_{{\left(\Lambda \cap \Omega \right)}^{c}}\left(f_{\theta }\left(\tilde{Y}\right)-Y_{0}-N\right)∥}_{2}^{2}|\tilde{Y}\right]=\nabla_{\theta }E\left[1_{q}^{T}M_{{\left(\Lambda \cap \Omega \right)}^{c}}|f_{\theta }\left(\tilde{Y}\right)-Y_{0}-N{|}^{2}|\tilde{Y}\right],$$

where $1_{q}$ is a *q*-dimensional vector of ones. Equation (32) from [25] shows that the conditional expectation of $f_{\theta }\left(\tilde{Y}\right)-Y$ on $M_{{\left(\Lambda \cap \Omega \right)}^{c}}$ and $M_{\Omega \setminus \Lambda }$ is related by a factor, P. By repeating that derivation with all instances of $f_{\theta }\left(\tilde{Y}\right)-Y$ trivially replaced with $|f_{\theta }\left(\tilde{Y}\right)-Y_{0}{-N|}^{2}$, a similar relationship can be derived for the latter, yielding the same P factor; see [25]. In brief, if the *j*th entry ${\tilde{Y}}_{j}$ is not zero, then the *j*th diagonal of $M_{\Omega \setminus \Lambda }$ is zero, so

(A17) $$\begin{array}{c} E\left[|M_{\Omega \setminus \Lambda }\left(f_{\theta }\left(\tilde{Y}\right)-Y_{0}-N\right){|}_{j}^{2}|{\tilde{Y}}_{j}\neq 0\right]=0. \end{array}$$

When the *j*th entry of ${\tilde{Y}}_{j}$ *is* zero,

(A18) $$E\left[|M_{\Omega \setminus \Lambda }\left(f_{\theta }\left(\tilde{Y}\right)-Y_{0}-N\right){|}_{j}^{2}|{\tilde{Y}}_{j}=0\right]=E\left[P_{jj}^{-1}|f_{\theta }\left(\tilde{Y}\right)-Y{|}_{j}^{2}|{\tilde{Y}}_{j}=0\right].$$

See (31) of [25] for a detailed derivation. By combining both cases from Equations (A17) and (A18), we obtain

(A19) $$E\left[|M_{\Omega \setminus \Lambda }\left(f_{\theta }\left(\tilde{Y}\right)-Y_{0}-N\right){|}_{j}^{2}|{\tilde{Y}}_{j}\right]=E\left[P_{jj}^{-1}|M_{{\left(\Lambda \cap \Omega \right)}^{c}}\left(f_{\theta }\left(\tilde{Y}\right)-Y_{0}-N\right){|}_{j}^{2}|{\tilde{Y}}_{j}\right].$$

By applying this result to Equation (A16) by multiplying with $1_{q}^{T}$ and bringing the masks outside the entry-wise magnitude, we obtain

$$\begin{array}{cc} \nabla_{\theta }E\left[1_{q}^{T}M_{{\left(\Lambda \cap \Omega \right)}^{c}}|f_{\theta }\left(\tilde{Y}\right)-Y_{0}-N{|}^{2}|\tilde{Y}\right]\; & =\nabla_{\theta }E\left[1_{q}^{T}PM_{\Omega \setminus \Lambda }|f_{\theta }\left(\tilde{Y}\right)-Y_{0}-N{|}^{2}|\tilde{Y}\right]\\ \; & =\nabla_{\theta }E\left[{∥P^{1/2}M_{\Omega \setminus \Lambda }\left(f_{\theta }\left(\tilde{Y}\right)-Y\right)∥}_{2}^{2}|\tilde{Y}\right], \end{array}$$

as required. □

To find the $\ell_{2}$ error of ${\hat{Y}}_{RSSDU}$, we use $M_{\Lambda \cap \Omega }+M_{{\left(\Lambda \cap \Omega \right)}^{c}}=1$ and the sum Equations (A13) and (A14):

$$\begin{array}{cc} \nabla_{\theta }E\left[{∥{\hat{Y}}_{RSSDU}-Y_{0}∥}_{2}^{2}|\tilde{Y}\right] & =\nabla_{\theta }E\left[{∥\left(M_{\Lambda \cap \Omega }+M_{{\left(\Lambda \cap \Omega \right)}^{c}}\right)\left({\hat{Y}}_{Nr2F}-Y_{0}\right)∥}_{2}^{2}|\tilde{Y}\right]\\ & =\nabla_{\theta }E\left[{∥\left(\frac{1+\alpha^{2}}{\alpha^{2}}M_{\Lambda \cap \Omega }+P^{1/2}M_{\Omega \setminus \Lambda }\right)\left(f_{\theta }\left(\tilde{Y}\right)-Y\right)∥}_{2}^{2}|\tilde{Y}\right] \end{array}$$

as required.

## Appendix D. Table of SSIM on Test Set

The mean SSIM of the magnitude images are shown in Table A1. The SSIM of the proposed methods is comparable to the fully supervised benchmark. However, in many cases, the methods that used BM3D outperformed even the fully supervised benchmark, implying that BM3D achieved a better SSIM than the machine learning-based approach to denoising used in this paper. We emphasize that the entirely data-driven approaches were not trained to minimize for the SSIM, and the SSIM would be expected to substantially improve if it was included in the loss function [36].

The methods that used BM3D had a considerably higher standard error, which indicates a substantially higher variation in the quality of the output. We believe that this was a consequence of the mismatch between BM3D’s Gaussian noise model and the actual error of the RSS estimate, which led to a higher risk of oversmoothing and artifacts such as those shown in Figure 5 and Figure 6.

**Table A1:** The methods’ test sets’ SSIM in the magnitude images with standard errors. The double lines separate the type of training data available and bold font is used to denote the best performance within each category. Asterisks denote proposed methods.

	Acceleration Factor $R_{\Omega }=4$				Acceleration Factor $R_{\Omega }=8$			
	$\sigma_{n}=0.02$	$\sigma_{n}=0.04$	$\sigma_{n}=0.06$	$\sigma_{n}=0.08$	$\sigma_{n}=0.02$	$\sigma_{n}=0.04$	$\sigma_{n}=0.06$	$\sigma_{n}=0.08$
Noisy and sub-sampled	0.76±0.006	0.64±0.006	0.50±0.006	0.40±0.005	0.72±0.008	0.67±0.007	0.57±0.006	0.48±0.006
Fully supervised benchmark	0.83±0.007	0.75±0.006	0.63±0.006	0.52±0.005	0.75±0.008	0.77±0.007	0.73±0.007	0.67±0.006
Supervised w/o denoising	0.83±0.006	0.70±0.006	0.55±0.005	0.43±0.005	0.80±0.008	0.74±0.006	0.63±0.006	0.52±0.005
Supervised with BM3D	0.86±0.025	0.75±0.042	0.64±0.043	0.55±0.041	0.85±0.014	0.78±0.033	0.69±0.039	0.61±0.039
Unweighted Noisier2Full *	0.83±0.007	0.75±0.006	0.63±0.006	0.52±0.005	0.76±0.008	0.77±0.007	0.73±0.007	0.66±0.006
Noisier2Full *	0.82±0.007	0.74±0.006	0.62±0.006	0.50±0.005	0.74±0.008	0.76±0.007	0.72±0.007	0.65±0.006
Standard SSDU	0.83±0.004	0.69±0.004	0.55±0.003	0.43±0.003	0.79±0.005	0.74±0.004	0.63±0.004	0.52±0.003
SSDU with BM3D	0.86±0.025	0.75±0.042	0.64±0.043	0.56±0.041	0.84±0.014	0.78±0.033	0.69±0.039	0.61±0.039
Noise2Recon-SS	0.83±0.006	0.71±0.006	0.56±0.005	0.47±0.005	0.79±0.008	0.73±0.006	0.66±0.006	0.56±0.005
Unweighted Robust SSDU *	0.83±0.007	0.75±0.006	0.62±0.006	0.51±0.005	0.75±0.008	0.77±0.007	0.72±0.006	0.65±0.006
Robust SSDU *	0.83±0.007	0.74±0.006	0.62±0.006	0.50±0.005	0.75±0.008	0.76±0.007	0.72±0.007	0.65±0.006
